# Measuring disparities to emergency medicine with 200 million voter records: The case of rural hospital closures

**DOI:** 10.1111/jrh.70019

**Published:** 2025-03-18

**Authors:** Michael E. Shepherd, Christian Cox, Derek A. Epp

**Affiliations:** ^1^ School of Public Health Health Management & Policy Department University of Michigan Ann Arbor Michigan USA; ^2^ Department of Economics University of Arizona Tucson Arizona USA; ^3^ Department of Government University of Texas at Austin Austin Texas USA

**Keywords:** hospital access, hospital closure, hospital distance, Medicaid expansion, racial/ethnic health disparities, rural health

## Abstract

**Purpose:**

Distance to health service providers is related to increased mortality risk and decreased service utilization. However, existing studies of distance to services often rely either on aggregated measures of distance or small samples of survey respondents. Nationwide individual data from 200 million Americans are used to assess various demographic groups’ distances to open acute hospitals.

**Methods:**

We gathered the exact location of every open acute hospital from the UNC Cecil G. Sheps Center and the Department of Health and Human Services. We merged this information with data on 200 million voters from the L2 voter file for 2020. We calculate each registered voters’ distance to the nearest open hospital in kilometers by demographic, region, and state Medicaid expansion status.

**Results:**

The average American adult is 5 miles from the nearest hospital. Native Americans and rural White Americans face the longest distances to medical services. Lower‐income adults face longer distances than higher‐income adults. Those over 65 are roughly 10% farther away in comparison to those 18‐40. Registered Republicans are 30% farther than registered Democrats. Recent hospital closures in states that have yet to expand Medicaid have contributed to all of these disparities.

**Conclusions:**

Lower‐income and older Americans, groups that tend to have worse health overall, face the longest travel distances to hospitals—perhaps contributing to income and age‐based health disparities. Native Americans and rural whites, who themselves experience considerable health hardship, also have significant travel burdens to receive hospital care. Registered Republicans have longer travels to emergency care than Democrats, adding to recent research on partisan health disparities.

## INTRODUCTION

Distance to medical services is associated with lower service utilization and higher risk of mortality.^1^, ^2^, ^3^, ^4^, ^5^, ^6^, ^7^, ^8^ From strokes and car crashes to COVID‐19, how far one happens to be from a hospital at the time of a medical crisis has important consequences. Making matters worse, many rural parts of the United States have lost their only local hospitals due to recent hospital closures, especially in states that have failed to expand Medicaid under the Affordable Care Act.^9^, ^10^ As a result, measuring accurately how far demographic groups are from hospitals reveals consequential information about health disparities and an important unfolding public health crisis. Despite this importance, studies measuring distance to services have relied upon aggregated geographic measures or smaller survey samples which each have limitations.

In the case of aggregate data, scholars have calculated how far the center of a county—or another geographic unit—is from the nearest medical provider (eg, Ref. 11). However, counties are uneven in size, with counties in the West and Midwest having larger land areas.^12^ Moreover, US counties exhibit considerable variation in their levels of demographic segregation.^13^, ^14^ Consequently, such estimates imprecisely account for the spatial distribution and distances to services of various demographic groups within geographies. Studies relying on samples of individuals (eg, Refs. 2, 3, 6, 15, 16) are limited in their ability to generalize outside of the smaller study setting—often a single city or region. Even studies relying on nationally representative or cross‐national survey data (eg, Refs. 6 and 15) often have too limited of samples from particular demographic groups to make reliable subnational inferences. These issues are especially large for understanding the effects of rural health access, where data in general but especially on rural subgroups are limited.

To improve our geographic and demographic understanding of rural health access, we merge data from the UNC Cecil G. Sheps Center^17^ and the Department of Health and Human Services on every open acute hospital with data on roughly 200 million voter records from the L2 voter file. L2 is a commercial vendor that complies and merges disparate sources of administrative data, like state voter registration lists and census records as well as consumer data. With these data, we measure each registered voter's distance in miles to the nearest hospital. This allows for a nationwide characterization of the distances adult US citizens from different demographic groups face in reaching a hospital that is more precise than any study to date. Moreover, these data allow us to incorporate information on distance by political partisanship for the first time ever, which has been documented to be of increasing importance in health outcomes—including COVID‐19 deaths, vaccines, following stay‐at‐home orders.^18^, ^19^, ^20^, ^21^ Finally and most importantly, with these data, we can explore more accurately than ever before how and how many rural US citizens of various groups have been affected by recent hospital closures.

We demonstrate that Native American citizens generally and those in rural areas face the longest distances to hospitals overall. Moreover, we show that those with incomes below $35,000 are 15% farther from services than those with incomes over $200,000, demonstrating important class disparities. Further, US citizens over the age of 65 live nearly 15% farther from emergency medical services than those below the age of 40. These findings reveal that individuals who are among the least healthy demographic groups in the United States are also those who are the farthest away from hospitals.^22^, ^23^ Reflective of regional partisan patterns and growing rural‐urban partisan polarization,^24^, ^25^ we find that registered Republicans are roughly 30% farther on average from hospitals than Democrats and nearly 20% farther than the national average. Importantly, Republicans live farther from hospitals even when we look at nonrural spaces.

We then turn to the distance disparities resulting from recent hospital closures.^9^, ^10^ We show that over 600k registered voters lost their nearest hospital between 2016 and 2020 alone. These losses have contributed to increased distances for nearly every demographic groups’ *national* average distance to emergency care. We show that nonexpansion state residents, lower‐income individuals, older individuals, and Republicans were more likely to experience hospital closures, but find limited evidence of racial‐ethnic differences in the likelihood of experiencing a closure.

We do, however, find large racial/ethnic distance disparities in the resulting distances to the next open hospital available for affected individuals in nonexpansion states. Black and Native American US citizens who lost their hospitals between 2016 and 2020 had resulting distances to the nearest open hospital that were over 40% higher than the national average and nearly 2 miles farther on average from the next open hospital than affected White US citizens. In expansion states where closures have become rarer, we show that even the handful of hospital closures that did occur between 2016 and 2020 did not lead to increased distances for affected voters, reflecting the closures’ more urban positioning and expansion states’ more robust health care options.

These patterns reveal that many US citizens, especially in rural areas, are dangerously far from hospital care. Exacerbating the problem of distance, the data imply that some of the most underserved and in need of health services on the basis of their demographic characteristics are among those who are the farthest away from emergency care. For those residing in states that have failed to expand Medicaid, rural hospital closures have aggravated the already considerable hospital distance problem.

## DATA AND METHODS

To more precisely measure distances, we enlist data from the University of North Carolina Cecil G. Sheps Center and the Department of Health and Human Services on the exact location (address number, street name, city, zipcode, and state) of every open acute hospital in the United States.^1^ After extracting this information, we merged these locations with the addresses of upwards of 200 million voters using the L2 voter files for the 2020 election.^2^ Voter files are statewide databases that contain information—such as name, address, registered party, whether the individual voted, age, and, depending on the state, sex, race, and ethnicity—on all individuals who are registered to vote.^26^, 3 Voter file data have become popular in political science for estimating the effects of various forces on voter turnout (eg, ^27^ and ^28^).

These data are largely publicly available but can be difficult to access, clean, or purchase. Commercial vendors have compiled these data, primarily for the purpose of selling data to political campaigns. Although states follow their own voter registration processes,^26^ L2 has a consistent set of information per state and supplements files with commercial datasets and census data where data is missing or not collected. L2's voter files cover practically all registered voters from 2016 to 2020, including those who register but never vote.

The files are structured as voter registration snapshots, meaning the current voter rolls at a given time. Individuals who are ineligible to vote, are purged, or have never registered are, therefore, absent from these data. The data are not equivalent to the voting‐age population or a census of all adults, but rather the voting‐eligible population. While voter file data are not perfect, existing errors tend to be small, random (eg, duplicate records, deceased individuals, and incorrect birth dates), and are likely unrelated to our quantities of interest.^26^, ^29^

We use the demographic information and the geolocated addresses of registrants from the 2020 file to calculate the Euclidean distance between where every registered voter in the US lives (n ≈ 200,000,000) and their closest open hospital. One may worry that straight‐line distances do not provide as reliable estimates of travel time as measures that incorporate traffic. Reassuringly, studies comparing measures of Euclidean distance and actual time in traffic have found that these 2 measures are essentially identical especially in rural areas, with some even expressing preference for the straight‐line measure.^30^, ^31^ Using USDA RUCA codes, we classify regions 1 (metropolitan area core) to 6 (micropolitan low community) as nonrural and 7 (small town core) to 10 (rural areas) as rural. There is no agreed‐upon standard, as some opt for metropolitan^1^, ^2^, ^3^ versus nonmetropolitan designations, while others utilize population density cutpoints or zipcode level data to measure “rural.”^12^, ^32^, ^33^

Another worry is that registered voters are not representative of the adult US population. If true, our measures of distance could be ungeneralizable—setting aside what that may imply about the status of American democracy.^34^, ^35^ However, political scientists have long noted that there are only minimal demographic and attitudinal differences between the voting and nonvoting populations.^36^, ^37^ On the basis of the characteristics available in the L2 voter file, there is considerable similarity between the characteristics of adults in the voter file and the entire US population. Table A1 demonstrates the representativeness of the voter file in comparison to data from the US Census.

The small differences that exist are largely driven by the lack of data in the voter file on individuals who are under 18 due to their ineligibility to vote. US citizens under 18 are more racially and ethnically diverse and less wealthy.^38^, ^39^, ^40^ This omission leads the percentages for racial and ethnic group composition, age, and income to differ by a few percentage points. Once accounted for, differences are negligible. Further, roughly 70% of eligible US citizen adults are registered to vote and all state registration rates fall between 60% and 83%, revealing considerable coverage across US states and regions.^4^ A huge number of US citizens (well over 50%) are represented in the voter files. Our largest lingering source of sample bias is the inability to capture noncitizen or immigrant distances to care.

We consider those who resided nearer to a closed hospital to have experienced a closure. In our regression analyses, we predict membership in this group (ie, having lost a hospital) using an ordinary least squares (OLS) regression (linear probability model [LPM]). We also include state‐level random effects. Fixed effects are inappropriate due to the inclusion of Medicaid expansion status. Some may question the use of an LPM given the binary outcome. LPMs can from time to time provide predictions that defy the 0‐1 bounds. However, econometricians have shown that the results from LPMs are easier to interpret (the average change in the probability of Y conditional on X), they tend to recover similar estimates to logistic regressions or other MLE models, and can even provide less‐biased estimates to probit or logit when fixed or random effects are incorporated.^41^, ^42^, ^43^ For the ease of interpretability without empirical loss, we opt for utilizing a LPM.

## NATIONWIDE RESULTS

We start in Table 1 by presenting the average distance in miles that US citizen adults of differing demographic groups must travel to access the nearest open hospital. The top row of data in the table reveals the overall US average distance and averages by region.^5^ The average US citizen adult is 5.05 miles (≈ 8 kilometers) away from the nearest open hospital. Those who live in the Northeast and West are closer to hospitals, while those in the Midwest and South are farther away. Overall, the Northeast has the smallest distance disparities across groups.

**TABLE 1:** Distance in miles to nearest open hospital in 2020.

	*US*		*Northeast*		*Midwest*		*South*		*West*	
	N (million)	Mean	N (million)	Mean	N (million)	Mean	N (million)	Mean	N (million)	Mean
Everyone	207.3	5.05	37.2	4.10	46.5	5.32	77.8	5.71	45.7	4.44
*Race*										
White	130.3	5.57	23.3	4.81	33.7	5.72	46.6	6.23	26.5	4.89
Black	21.6	4.06	3.1	1.86	3.5	2.72	13.8	5.00	1.1	2.83
Hispanic	25.0	3.92	3.9	2.48	2.2	4.10	9.4	4.67	9.3	3.74
Asian	7.0	3.48	1.4	2.73	0.8	4.19	1.7	4.34	3.0	3.13
Native American	0.2	9.14	0.004	5.04	0.01	7.41	0.1	8.03	0.04	13.88
Other	4.9	3.82	1.2	3.13	1.0	4.18	1.2	4.44	1.4	3.66
*Political party*										
Democrat	83.9	4.36	16.1	3.36	17.0	4.40	32.2	5.13	18.4	3.84
Republican	64.0	6.00	9.1	5.19	14.8	6.38	27.1	6.44	12.8	5.23
*Income*										
*<* 35k	23.2	4.87	4.0	3.34	5.7	4.81	9.7	5.66	3.7	4.59
35k‐99k	105.7	5.27	16.3	4.22	25.8	5.51	41.9	5.97	21.6	4.42
100k‐199k	55.9	4.35	11.6	4.30	11.20	5.21	18.5	5.28	14.5	4.12
200k+	16.7	4.23	4.2	3.72	2.6	4.72	5.4	4.78	4.4	3.76
*Age*										
18‐26	24.9	4.78	4.1	3.92	5.0	5.13	9.4	5.55	6.2	3.93
27‐40	47.3	4.67	8.5	3.69	10.1	5.03	17.6	5.40	11.0	3.97
41‐64	79.7	5.17	14.6	4.21	17.7	5.48	30.2	5.84	17.0	4.48
65+	55.2	5.33	9.8	4.38	13.5	5.37	20.5	5.88	11.3	5.10

*Note*: Figure 1 provides the straight‐line distance in miles to the nearest open hospital.

*Sources*: UNC Cecil Sheps Center^17^; L2 Voter File.

Native American and White US citizens live farther on average in every region of the United States than other groups. Native American US citizens live an average of 9.14 miles from hospitals or about 60% farther than the national average. White US citizens overall live 10% farther than the national average and between 15% and 20% farther than average in the more rural South and Midwest. Asian, Black, and Hispanic adults face the shortest distances on average, reflecting the relative density of these groups in urban centers with more medical providers.^13^, ^14^

Table 1 shows that wealthier individuals and younger people^18^, ^19^, ^20^, ^21^, ^22^, ^23^, ^24^, ^25^, ^26^, ^27^, ^28^, ^29^, ^30^, ^31^, ^32^, ^33^, ^34^, ^35^, ^36^, ^37^, ^38^, ^39^, ^40^ live considerably closer to hospitals than lower‐income and older groups, with the largest income and age distance disparities occurring in the South and West regions. In comparison to those with incomes over $200,000, those with incomes less than $35,000 live about 15% farther from hospitals. In terms of age, people aged 27‐40 are 13% closer to hospitals than those over the age of 65. Age disparities in proximity to hospitals are largest in the West, where those over 65 are 25% farther than those under 40.

As a consequence of the urban clustering of racial and ethnic minority groups, these groups’ high rates of Democratic partisanship,^14^, ^44^ and broader rural‐urban partisan sorting,^24^, ^25^ there are large partisan differences in proximity to hospitals. The average registered Republican is 31.6% farther from hospitals than the average registered Democrat. Similar to the large partisan COVID‐19 fatality and COVID prevention behavior disparities observed by others,^19^, ^21^ partisan distance disparities exist in every region of the United States.

Even in the Northeast, which as we have observed has lower overall distance disparities, Republicans live 43% farther away than Democrats. These partisan differences imply that some of the partisan COVID‐19 and health disparities observed in previous research are likely partially driven by these distance disparities. Importantly, these partisan differences are not exclusively rural‐urban differences, as Republicans tend to live farther even in less rural areas.

Table 2 compares the distances of all of these groups among those living in rural versus non‐rural areas. These trends reveal larger rural‐urban distance disparities across all groups. Rural residents are about 70% farther away from hospitals than non‐rural residents and 60% farther than the nationwide average. Moreover, some of the national demographic disparities observed previously are apparent in rural, with older rural US citizens facing the largest distances to hospitals as well as Native and Hispanic rural US citizens facing the farthest distances to hospital care overall.

**TABLE 2:** Distance in miles to nearest open hospital in 2020, rural versus non‐rural.

	*Rural*		*Non‐rural*	
	N (millions)	Mean	N (millions)	Mean
Everyone	14.8	9.54	192.5	4.71
*Race*				
White	11.7	9.40	118.6	5.19
Black	0.8	8.45	20.7	3.89
Hispanic	0.6	11.30	24.3	3.73
Asian	0.1	9.74	6.9	3.39
Native American	0.03	16.62	0.1	7.50
Other	0.1	11.52	4.8	3.64
*Political party*				
Democrat	4.4	9.34	79.5	4.08
Republican	6.3	9.46	57.7	5.63
*Income*				
*<* 35k	2.2	9.48	21.0	4.38
35k‐99k	9.1	9.09	96.6	4.91
100k‐199k	1.8	9.12	54.0	4.61
200k+	0.4	9.22	16.3	4.10
*Age*				
18‐26	1.3	4.93	23.5	4.54
27‐40	2.6	9.09	44.6	4.41
41‐64	5.6	9.47	74.1	4.84
65+	5.1	10.00	50.1	4.85

*Note*: Table 2 provides the straight‐line distance in miles to the nearest open acute hospital for individuals living in rural and non‐rural regions.

*Sources*: UNC Cecil Sheps Center^17^; L2 Voter File.

## HOSPITAL CLOSURE RESULTS

We apply these data to the unfolding rural hospital closure crisis.^5^, ^7^, ^9^, ^10^, ^45^, ^46^ Since the 1980s, dozens of rural hospitals have closed across the United States. As a result, many US citizens are now perilously far from hospitals. Scholars have found rural hospital closures lead to longer overall distances to care, slower emergency medical service (EMS) response times, and increased mortality risk.^47^

Medicaid expansion through the Affordable Care Act (ACA) has altered patterns of rural hospital closures. Specifically, Medicaid expansion has lowered the numbers of uninsured individuals living in the states that chose to expand, especially in rural communities within these states.^48^, ^49^, ^50^ However, for the largely Republican states that chose not to expand Medicaid,^51^ health insurance coverage gaps persisted and in some cases worsened.^52^ For hospitals in states that expanded Medicaid, these reduced uninsured populations meant fewer uninsured people arriving at hospitals to receive free care and, thus, lowered uncompensated care loads for hospitals and reduced their risk of closure. For hospitals operating in nonexpansion states, the risks of closure worsened with continued rising rates of uncompensated care (eg, Refs. 47, 53, 54).

**FIGURE 1:**
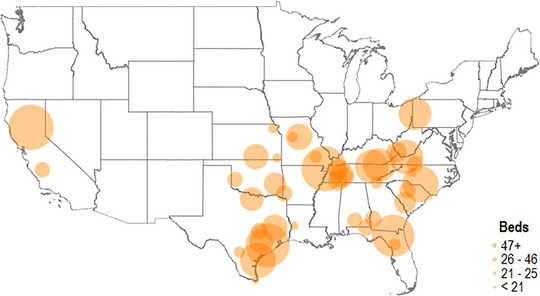
Location of hospital closures, 2016‐2020. *Note*: Figure 1 provides the geographic location of hospital closures that occurred between 2016 and 2020. *Source*: UNC Cecil G. Sheps Center.^17^

Figure 1 plots the location of hospital closures that occurred between 2016 and 2020 with UNC Sheps Center data^17^ to highlight these realities. Following definitions outlined by the Sheps Center, we consider a hospital to be closed if the facility has ceased to provide “general, short‐term, [or] acute impatient care.”^6^ We did not consider “converted” hospitals, hospital mergers, or those that only closed for a limited period of time as closed. We plot the size of each dot representing each closure in Figure 1 based on the number of hospital beds lost as a result of the closure. Overall, 625,000 registered voters, representing an estimated 1,000,000 US citizens adults based on voter registration rates, lost their hospital between 2016 and 2020.

**FIGURE 2:**
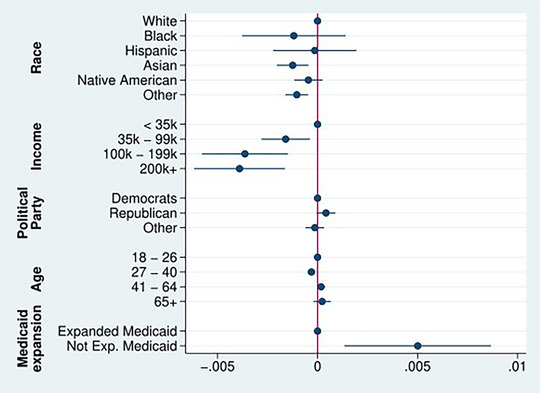
Coefficients characterizing the probability of having the nearest hospital close between 2016 and 2020. *Note*: Model includes state‐level random effects and robust standard errors. Reference categories are White, < 35k, Democrats, 18‐26, and Medicaid expansion. See Table A2 for full results.

Figure 1 reveals that recent closures have concentrated in the Southern and Midwestern states that have failed to expand Medicaid. To formally probe the role of Medicaid expansion and explore demographic differences, Figure 2 characterizes the probability of an individual having their nearest hospital close as a function of their demographics and whether they lived in a state that had yet to expand Medicaid as of 2020, as nonexpansion states have come to experience higher closure rates.^10^ Rather than relying on estimated treatments from counties,^46^, ^55^, ^56^ we determine whether a person was affected by a hospital closure by calculating each individual's distance to the nearest open hospital and the nearest closed hospital. Thus, we are able to consider a broader set of individuals as having lost their closest hospital, how the likelihood of experiencing a closure varies across subgroups, and estimate how the resulting “next” hospital is for affected people for the first time. Figure 2 plots the results of this analysis.

Although hospital closures are rare overall, Figure 2 reveals that lower‐income individuals, those over 40, and Republicans (core rural groups) are far more likely to have experienced them recently. However, these demographic differences are dwarfed by the political differences arising from Medicaid expansion status. Overall, about 80% of adults affected by hospital closures between 2016 and 2020 were in nonexpansion states. Living where Medicaid has not been expanded increases the probability one will experience a local hospital closure by 0.5 percentage points.

Table 3 compares the distances registered voters whose nearest hospital closed now must travel to get to the nearest still‐open hospital. Those who were affected by local hospital closures in nonexpansion states averaged distances of almost 7 miles to the nearest open hospital following a closure, representing a 31% increase over the national average.

**TABLE 3:** Mean miles to still‐open hospital for those whose nearest hospital closed.

	*US*		*Not expanded*		*Expanded Medicaid*	
	N	Mean	N	Mean	N	Mean
Everyone	624,993	6.14	495,701	6.91	129,292	3.31
*Race*						
White	428,730	6.04	320,899	7.05	107,831	3.15
Black	70,585	7.91	67,868	8.15	2,717	2.93
Hispanic	80,773	5.23	73,298	5.08	7,475	7.04
Asian	3,530	6.40	2,720	7.34	810	3.33
Native American	944	7.86	910	8.09	34	2.52
Other	3,998	5.45	3,018	6.47	980	2.41
*Political party*						
Democrat	238,476	5.91	192,091	6.57	46,385	3.30
Republican	256,476	6.12	203,723	6.97	52,402	2.95
*Income*						
*<* 35k	121,350	6.19	99,912	6.82	21,438	3.38
35k‐99k	386,562	6.19	304,808	6.94	81,754	3.48
100k‐199k	68,040	5.97	54,182	6.80	13,858	2.86
200k+	14,819	5.59	11,827	6.36	2,992	2.69
*Age*						
18‐26	72,962	6.12	59,142	6.69	13,820	3.76
27‐40	124,900	6.02	99,171	6.81	25,729	3.10
41‐64	243,142	6.15	192,118	6.96	51,024	3.21
65+	183,989	6.22	145,270	6.99	38,719	3.44

*Note*: Table 3 provides the straight‐line distance in miles to the nearest open acute hospital for individuals affected by a local hospital closure.

*Sources*: UNC Cecil Sheps Center^17^; L2 Voter File.

Table 3 reveals that for the remaining 20% residing in expansion states, the distance to the nearest replacement hospital was not as large (3.3 miles). In Table A3, we show that partially as a consequence of these closures, the *national* average distance to hospitals increased by 0.2% from 2016 to 2020. Some demographic groups had even higher rates of increased distance during this time.

Once affected by a closure in a nonexpansion state, essentially all demographic groups had longer distances to the nearest still‐open hospital than their respective groups’ national average. However, because certain groups are more likely to live farther from hospitals generally even within a county—aggregate measures of resulting distances following closures assume identical subgroup distances despite these large underlying demographic distance differences. Table 3 shows our improvements on these marks. We show that Black US citizen adults experiencing local hospital closures in nonexpansion states must travel twice as far in comparison to the national average from Black US citizens and nearly 1.1 miles farther than rural White US citizens who lost their hospital in these same states. Native American citizens were also about 1 mile farther than similarly affected White US citizens. Though rural White US citizens constitute about 65% of the people experiencing nonexpansion state hospital closures, rural Black and Native American US citizens bear the brunt of the distance chances from them.

Outside of race and ethnicity, rural hospital closures have made hospital care farther out of reach for older and lower‐income US citizens. Those over 65 represent nearly a one‐third of those affected by hospital closures in nonexpansion states. Additionally, adults over the age of 65 now face travel distances roughly 30% greater than adults of the same age average nationally. Those with incomes lower than $35,000 in nonexpansion states were nearly 35% farther from the nearest open hospital than other lower‐income adults overall and about 0.5 miles farther in travel than individuals making over $200,000 who also experienced closures.

In other words, lower‐income and older populations—people who depend on hospitals for medical care more often despite already living farther from them^57^, ^58^, ^59^—also face the longest differences after closures in nonexpansion states.

Partisan proximity differences were again evident. Registered Democrats affected by closures in nonexpansion states must travel an average 6.57 miles to the nearest open hospitals, considerably farther than the 4.36 national average for Democrats. Registered Republicans in nonexpansion states must travel an additional 5% farther than registered Democrats who also experienced closures in nonexpansion states.

Just over half a million adult rural voters across the country in 2020 were affected by a hospital closure. Exacerbating these effects, an additional 31,000 people (or 5% of hospital closure victims) also had their second closest hospital close during this time, forcing this group to travel even farther to their closest open hospital.

Among the adults affected by 2 closures, the next closest open hospital was an average of an additional 15 miles away. These distances increase drastically as affected individuals must chose between the set of open hospitals available to them once the first or second closest hospital closes. We find that the average second, third, and fourth closest options for those who lost their rural hospital ranged from 15 to 25 miles away, respectively, all greatly increasing travel burdens for rural US citizens in need of hospital care. Those affected by rural closures are especially vulnerable as they already have larger distances to begin with. The average distance to the closest hospital among the 206,687,576 individuals unaffected by closures was 5.02 miles, with their second closest being 9.66 miles away and third closest 12.94 miles. Thus, by implication, the millions unaffected by closures have 3 hospital options that are closer than any of the available options for those who have lost their local hospital.

In addition to increases in distance, the hospitals remaining open to serve those who have lost their nearest hospitals will likely experience sizable increases in demand in the coming years. For closed hospitals, the average number of hospital beds previously available was 36 and the estimated number of beds per patient (based on those affected by the closure) was 673. But when a hospital closes, the nearest still‐open hospital must accommodate new patients. Assuming that patients affected by a closure converge on their second‐nearest hospital, we estimate that closures led to just under an additional 4000 new adults seeking care in the next‐nearest hospital, increasing patients per bed for these hospitals by 76 (14%).

## DISCUSSION AND CONCLUSION

We merge the location of every open hospital and the addresses of nearly 200 million registered voters. With these data, we are able to provide the most accurate measures of distance disparities to emergency medicine across demographic groups, states, and regions in the United States to date. We show that lower‐income and older adults as well as Native Americans, Whites, people in rural areas, and Republicans tend to live the farthest from care.

Applying these data to an important unfolding health issue, we explore the rural hospital closure crisis. We show that the strongest predictor of recent hospital closures is residing in states that have not expanded Medicaid. Further, these closures in nonexpansion states have led to sizable increases in travel distances for affected voters from essentially every studied demographic group. Lower‐income, older, and Black US citizens in the rural South have experienced the largest increases in percentage terms among demographic groups. In comparison to the national average, adults who lost their local hospital in nonexpansion status were an average of 1.9 miles farther away.

These findings have important contributions to the study of health access in the United States. First, because of the size of the voter file, we are able to provide more accurate and robust subnational measures of distances to care than previous studies, especially in rural areas. As a result, we are able to highlight that some of the least healthy and most marginalized social groups in the United States. (eg, those over 65, those with lower incomes, rural US citizens) are often the farthest away from medical attention. We also add to work on growing partisan differences in health. In addition to partisan differences in ACA participation,^50^ COVID‐19‐related outcomes, and prevention behaviors,^19^, ^21^ we demonstrate large partisan differences in proximity to hospitals.

Despite these advances, our work is limited in important respects. We lack data on people under the age of 18 and US citizens. As a result, we cannot speak to the distance disparities faced by children or noncitizen populations across the United States. Additionally, our work can only speak to distance to care as a form of disparity. We cannot document how other factors may exacerbate the effects of distance. Future research may merge other administrative health data or data on the locations of other types of providers with the voter file to explore how policies influence service proximity and the effects of distance on other types of health outcomes.

## CONFLICT OF INTEREST STATEMENT

The authors report no conflicts of interest.

## Data Availability

The data that support the findings of this study are available from L2 Voter File. Restrictions apply to the availability of these data, which were used under license for this study. Data are available from the author(s) with the permission of L2 Voter File.

## END NOTES

1 We do not include Children's, military/VA, psychiatric, or indigenous hospitals (on reservations or operated by the IHS). Only 13% of Native American citizens live on reservations, so most are covered by the hospitals in our sample.

2 The L2 data was accessed via Yale University servers [as part of a 2023 project] using an author's Yale sponsored identity access.

3 States are required to compile this information due to the “Help America Vote Act.”

4
https://www.census.gov/newsroom/press‐releases/2023/2022‐voting‐registration.html; https://www.kff.org/other/state‐indicator/number‐of‐voters‐and‐voter‐registration‐inthousands‐as‐a‐share‐of‐the‐voter‐population

5 Northeast: Connecticut, Maine, Massachusetts, New Hampshire, Rhode Island, Vermont, New Jersey, New York, and Pennsylvania. Midwest: Indiana, Illinois, Michigan, Ohio, Wisconsin, Iowa, Nebraska, Kansas, North Dakota, Minnesota, South Dakota, and Missouri. South: Delaware, District of Columbia, Florida, Georgia, Maryland, North Carolina, South Carolina, Virginia, West Virginia, Alabama, Kentucky, Mississippi, Tennessee, Arkansas, Louisiana, Oklahoma, and Texas. West: Alaska, California, Hawaii, Oregon, Washington, Arizona, Colorado, Idaho, New Mexico, Montana, Utah, Nevada, and Wyoming.

6
https://www.shepscenter.unc.edu/programs‐projects/rural‐health/rural‐hospitalclosures

## APPENDIX A

### A.1 Descriptive statistics

**TABLE A1:** Comparing demographics of those in L2 voter file to 2020 census.

	*2020 Voter file*		*Census*	
	N (millions)	% Total	N (millions)	% Total
Everyone	207.3	100	331.4	100
*Race*				
White	130.3	68.9	235.4	71.0
Black	21.6	11.4	46.9	14.2
Hispanic	25.0	13.1	62.0	18.7
Asian	7.0	3.7	24.0	7.2
Native American	0.2	0.1	9.6	2.9
Other	4.9	2.6	49.9	15.1
*Household income*				
*<* 35k	23.3	11.5	77.2	23.3
35k‐99k	106.1	52.4	129.5	39.1
100k‐199k	56.0	27.7	84.8	25.6
200k+	16.7	8.2	39.4	11.9
*Age*				
0‐17	0	0	73.4	22.1
18‐26	24.9	12.0	40.0	12.0
27‐40	47.5	22.8	63.1	19.0
41‐64	79.9	38.4	99.9	30.1
65+	55.4	26.6	54.8	16.5

### A.2 Regression results

**TABLE A2:** Regression predicting likelihood of having nearest hospital close.

	Coefficient (SE)
White	–
Black	−0.001 (0.001)
Hispanic	−0.000 (0.001)
Asian	−0.001*** (0.000)
Native American	−0.000 (0.000)
Other	−0.001*** (0.000)
Democrat	−
Republican	0.000* (0.000)
Other	−0.000 (0.000)
*<* 35k	−
35k‐99k	−0.001** (0.000)
100k‐199k	−0.003*** (0.001)
200k+	−0.003*** (0.001)
18‐26	−
27‐40	−0.000*** (0.000)
41‐64	0.000* (0.000)
65+	0.000 (0.000)
Expanded Medicaid	−
Not expanded Medicaid	0.005*** (0.001)
Constant	0.003*** (0.000)
Observations	184,188,049

*Note*: Regression includes state‐level random effects.

*** *P* < .01.

** *P* < .05.

* *P* < .10.

### A.3 Trends

**TABLE A3:** Distance in miles to nearest open hospital 2016‐2020.

	*2016*		*2018*		*2020*	
	N (millions)	Mean	N (millions)	Mean	N (millions)	Mean
Everyone	182.8	5.04	183.9	5.02	207.3	5.05
*Race*						
White	118.3	5.53	117.8	5.53	130.3	5.57
Black	19.2	4.00	19.3	4.00	21.6	4.06
Hispanic	19.7	3.82	21.0	3.85	25.0	3.92
Asian	5.3	3.43	5.7	3.44	7.0	3.48
Native American	0.1	9.62	0.1	9.12	0.2	9.14
Other	3.9	3.79	4.1	3.79	4.9	3.82
*Political party*						
Democrat	71.5	4.44	71.2	4.40	83.9	4.36
Republican	58.3	5.85	57.9	5.91	64.0	6.00
*Income*						
*<* 35k	26.1	4.55	25.0	4.84	23.2	4.87
35k‐99k	106.4	5.19	100.6	5.21	105.7	5.27
100k‐199k	35.8	4.49	43.2	4.67	55.9	4.76
200k+	6.3	4.05	9.5	4.15	16.7	4.23
*Age*						
18‐26	21.0	4.71	20.3	4.72	24.9	4.78
27‐40	40.2	4.67	40.2	4.63	47.3	4.67
41‐64	74.8	5.17	73.6	5.15	79.7	5.17
65+	46.6	5.28	49.7	5.27	55.2	5.33
